# Exploring power dynamics and their impact on intraprofessional learning

**DOI:** 10.1111/medu.14706

**Published:** 2021-12-13

**Authors:** Natasja Looman, Tamara van Woezik, Dieneke van Asselt, Nynke Scherpbier‐de Haan, Cornelia Fluit, Jacqueline de Graaf

**Affiliations:** ^1^ Department of Primary and Community Care Radboudumc Nijmegen The Netherlands; ^2^ Department of Geriatric Medicine Radboudumc Nijmegen The Netherlands; ^3^ Department of General Practice and Elderly Care University Medical Centre Groningen The Netherlands; ^4^ Department for Research in Learning and Education Radboudumc Health Academy Nijmegen The Netherlands; ^5^ Department of Internal Medicine Radboudumc Health Academy Nijmegen The Netherlands

## Abstract

**Background:**

During postgraduate training, considerable efforts for intraprofessional education are in place to prepare primary care residents (PC residents) and medical specialty residents (MS residents) for intraprofessional collaboration (intraPC). Power dynamics are inherently present in such hierarchical medical contexts. This affects intraPC (learning). Yet little attention has been paid to factors that impact power dynamics. This study aims to explore power dynamics and their impact on intraPC learning between PC residents and MS residents during hospital placements.

**Methods:**

This study expands on previously published ethnographic research investigating opportunities and barriers for intraPC learning among residents in five Dutch hospitals. We analysed transcripts of observations and in‐depth interviews using template analysis. A critical theory paradigm was employed. Discourse analysis additionally informed the data.

**Results:**

We defined five interrelated themes that describe characteristics of power dynamics in intraPC learning during hospital placements: beliefs; power distribution; interaction style; subjection; and fearless learning. Power dynamics operate both within and between the themes: power distribution between PC residents, MS residents and MS supervisors seemed to be an attribution affected by underlying beliefs about professional norms or about other professions; beliefs influenced the way PC residents, MS residents and MS supervisors interacted; power distribution based on inequity could lead to subjection of PC residents; power distribution based on equity could lead to fearless learning; and open interactions enabled fearless intraPC learning.

**Conclusions:**

Power dynamics have an impact on intraPC learning among residents in hospitals. Constructive power dynamics occur when power distribution is based on equity, combined with sincere open interactions, actively inviting each other into discussions and enlisting the support of MS supervisors to foster fearless learning. This can be achieved by creating awareness of implicit beliefs and making them explicit, recognising interaction that encourages intraPC learning and creating policies that support fearless intraPC learning.

## Abstract 2

This article demonstrates how residents' beliefs/attitudes affect their interactions with each other. The results suggest effective change can be achieved by small interventions that are easy to implement.

## 1 INTRODUCTION

Collaborative practice between primary care (PC) physicians and medical specialists (MSs) is vital and requires mutual trust and respect.^1^, ^2^, ^3^, ^4^ In the deep‐rooted hierarchical contexts of hospitals, however, it could be a measure of status for MSs to disrespect lower‐status professionals with impunity,^5^ such as PC physicians.^3^, ^6^, ^7^ Power dynamics based on traditional hierarchies are inherently present in (intra)professional interaction and learning processes^5^, ^6^, ^8^, ^9^, ^10^ and could have an adverse effect on collaborative practices^5^, ^8^ leading to adverse events in healthcare.^3^, ^11^ Often power dynamics are not openly discussed, but referred to implicitly, contributing to the hidden curriculum.

To prepare PC residents (PC residents) and medical specialty residents (MS residents) for collaborative practice, the learning of intraprofessional collaboration (intraPC) through intraprofessional education (intraPE) is an emerging part of postgraduate training.^12^, ^13^, ^14^, ^15^, ^16^, ^17^, ^18^ For example, hospital placements, where PC residents and MS residents work together at the same department, provide several opportunities for intraPE.^15^ These placements occur worldwide.^15^, ^19^, ^20^, ^21^, ^22^, ^23^ A Dutch study found that PC residents, MS residents and MS supervisors mentioned issues with power dynamics that influenced intraPC learning during hospital placements.^15^ Arabic studies have found that the personal attitude of MSs can make PC residents experience inferiority of feel inferior, leading to deficiencies in learning during hospital placements.^24^, ^25^ Canadian studies, furthermore, have found that more than one‐third of the PC residents experience harassment and intimidation arising from power dominance by MSs and MS residents during hospital placements.^26^, ^27^ As such, power dynamics can lead to interpersonal fear.^28^

Although considerable efforts are being made to design interprofessional/intraprofessional education (IPE/intraPE), little attention has so far been given to factors that impact hierarchy and power dynamics.^8^, ^29^ The vast majority of studies about IPE/intraPE focus on programmes or curricula, but omit to critically investigate the impact of power.^8^, ^30^ The same holds true for studies about hospital placements. By not addressing power dynamics, however, an ambiguous and opaque problem remains in place.^30^, ^31^ To improve the learning climate for intraPC learning, PC residents, MS residents and their supervisors need to have a better understanding of the impact of power dynamics.^8^

## 2 THEORETICAL BACKGROUND

In scientific literature, power and power dynamics seem to be easier to recognise than to define. Dahl (1957) explains power as a form of control: ‘A has power over B to the extent that he can get B to do something that B would not otherwise do’.^32^ A/B can be a person, team or organisation. King Jr (1968) describes power as the ability to bring about change^33^ or as the capacity to act or not to act. Raven (2010) defines power as a form of interpersonal influence which may be based on various sources: expertise, information, (formal) position, being a reference or the ability to exert coercion or reward.^34^ Bynum (2021), finally, elaborates that power hierarchies/distribution in medical learning environments are often manifested through knowledge, vulnerability, risk taking and influence.^10^

Underlying these definitions are philosophical roots of thinking about power. Arendt (1970) and Foucault (1976) explain that there is not one place or person where power emerges from, but that it is rather constructed between people and continues to exist as long as these people stay together.^35^, ^36^ The interaction of power between people can be understood as a dynamic process,^35^, ^37^ as an unstable network of practices that spreads throughout society and may exist within workplaces, institutions, or other places where people come together. In this article, we use the term ‘power dynamics’ to describe the way in which power impacts the interaction of two or more people or groups. Power and power dynamics are essentially neutral, not necessarily negative,^36^, ^38^ and its manifestation and impact may be constructive or non‐constructive.

Prior research demonstrates that the impact of power dynamics between higher status and lower status individuals may be moderated by psychological safety and perceived connectedness.^8^ Edmondson defines psychological safety as the extent to which people view the work/learning environment as being conducive to interpersonal risk‐taking, such as expressing themselves or asking for help, without fear of negative consequences.^7^, ^39^ It has been shown that an unconstructive manifestation of power dynamics can be overcome with high psychological safety, even in contexts with strong hierarchies.^40^, ^41^

### 2.1 Research aim

The aim of this study is to explore power dynamics and their impact on intraPC learning between PC residents and MS residents during hospital placements. The intention here is to enhance the understanding of the nature and extent of power dynamics on hospital wards and to pave the way for future constructive collaborative learning and practice.

## 3 METHODS

### 3.1 Context and design

Worldwide, during postgraduate training, PC residents undertake hospital placements in the same departments where MS residents are in training.^15^, ^19^, ^20^, ^21^, ^22^, ^23^ In the Netherlands, this means that PC residents work four days a week on the hospital ward together with MS residents; the fifth day is spent with other PC residents at the PC specialty training institute. This current study expands on previously published research by Looman et al. (2020), which investigated opportunities and barriers to intraPC learning between PC and MS residents during hospital placements.^15^

### 3.2 Data collection

In our previous study, observations and interviews were conducted at three geriatrics departments and three emergency departments of five Dutch hospitals from February to May 2018. During this study, issues of power and power dynamics repeatedly surfaced in interviews, even when power was not initially addressed by the interviewer. After 15 interviews, we decided to incorporate additional questions to explore this issue deeper in the subsequent 27 interviews. Previous studies on psychological (un)safety in healthcare have recommended taking different power status levels into account, and involving the researcher as an observer in the study setting to observe patterns rather than relying on participants' reports only.^42^ We finally used all 42 interviews for this study and included 24 fieldnote transcripts for triangulation. More information on data collection can be found in Looman et al. (2020).

### 3.3 Design

We decided that the issue of power dynamics needed another theoretical framework than the prior study on opportunities and barriers to intraPC. Due to the current focus on power dynamics and the sensitivity required for such a topic, we employed a critical theory paradigm. Critical theory is a research paradigm that focuses on the experience of people and seeks to understand how social structures shape these experiences.^43^, ^44^ Critical theory is concerned with issues such as power and justice and tries to explain how social systems function by looking into discourses, ideologies and institutions.^43^, ^45^ In line with this paradigm, a discourse analysis approach informed our data analyses.^45^, ^46^ Discourse analysis focuses on the relation between language, practice and power^46^ and assumes that it is important to analyse power relations from the viewpoint of the participant.^44^

### 3.4 Data analysis

Transcripts of the interviews and fieldnotes were analysed employing a template analysis method.^47^, ^48^ Template analysis can be accommodated to different paradigms,^49^ in this case critical theory and some discourse analysis elements as an additional way of looking at the data.^46^ For example, we used mental models and metaphors to analyse the data on a deeper level.^44^ Mental models show what people believe about others.^44^ Metaphors can reveal beliefs or norms that are normally hidden. We used mental models and metaphors as a discourse analysis approach to explore the power dynamics in our transcripts and to identify implicit forms of power.

Our data analysis started by selecting the relevant material. We combined an inductive and a deductive approach for the operationalization of power dynamics. Two authors (NL and TW) performed a first round of open coding. NL and TW each independently coded three transcripts. We discussed the results together. Combining these with sources in the literature, we made a preliminary template of power dynamics. We used the preliminary template to select relevant parts of the other transcripts. After that, NL and TW coded six transcripts individually and compared the similarities and differences. Due to different professional backgrounds, we had to settle on some definitions. ‘Team dynamic’, for instance, was coded when it was negative by NL, whereas TW interpreted it as neutral. We agreed to use it as a negative term and to use work‐climate as a neutral or positive term. NL and TW made an initial template and discussed this with the extended team: CF, NS and JdG.

In the second round, NL and TW divided and coded the remaining transcripts individually. Six of the transcripts were again coded by both and discussed in weekly meetings, to keep track of differences and similarities. We discussed and settled on differences by meeting with the whole research team and resolved all inconsistencies through consensus. Differences mainly concerned whether a quote was to be interpreted as neutral or negative, or how to choose a slightly different subcode from a larger overarching category (e.g., *hegemony* or *distance*). Other differences could be traced back to the different backgrounds of the researchers, in which case we opted for an inclusive approach and kept both codes (e.g., *collaboration* and *work‐climate*).

Finally, we double coded the fieldnotes and triangulated these with the findings in the coding template. We looked for mentions of power in the fieldnotes and compared these to what the interviewees had said.

### 3.5 Reflexivity

NL is a psychologist and PhD candidate in intraPC/intraPE. Working as an psychologist, her focus is on the underlying aspects of behaviour, interaction and equity between people in a work environment. TW has a background in education science and philosophy. She is a teacher trainer and researcher in medical education. She holds an enactivist approach to learning, focusing on the role of affect and environment in learning. DvA is a geriatrician, supervisor and researcher in medical education. She focuses on team behaviour in the hospital ward regarding intraPC learning between residents. NS is a general practitioner, director of PC specialty training and professor general practice in IPC. Her focus is on the role of PC residents with regard to intraPC learning. CF is an MD and educationalist and professor of workplace learning. Her focus is on creating working environments that stimulate learning for both students and professionals, psychological safety and adaptive expertise. JdG is an internist, director of postgraduate medical education and professor of professional performance in PGME. She focuses on hierarchy, psychological safety and policies that affect intraPC learning.

## 4 RESULTS

Based on our analysis, we defined five interrelated themes that describe characteristics of power dynamics in intraPC learning between PC residents and MS residents during hospital placements: (i) beliefs; (ii) power distribution; (iii) interaction style; (iv) subjection; (v) fearless learning (see Table 1).

**TABLE 1:** Themes that describe characteristics of power dynamics in intraPC learning between primary care (PC) residents and medical specialty (MS) residents in hospitals

Theme	Description
A. Beliefs	Participants hold certain beliefs about other professions (mental model of the other) or about existing power systems and standards (professional norms). This concerns beliefs between PC and MS residents and between residents and MS supervisors in hospitals.
B. Power distribution	Power distribution between PC physicians/PC residents, MSs/MS residents and MS supervisors appears to be an attribution and can be based on systems in the organisation. Power can be attributed, for instance, as hierarchical status due to mastery of knowledge. Power distribution is part of a system as an existing power distance between medical disciplines (PC and MS) and between supervisors and residents. Power distribution appears to be an intertwining of attribution and system factors, such as a skewed power distance in which MSs/MS residents have a superior and PC physicians/PC residents an inferior hierarchical/power status (hegemony). The distribution of power can be based on either equity or inequity.
C. Interaction style	Power is expressed in how participants talk about and with each other, what words they use (metaphors, communication style) and whether the interactions are open and collaborative.
D. Subjection	Subjection is a type of behaviour of PC residents in terms of not taking interpersonal risks or withdrawal and ceasing engagement. These behaviours can occur in a dependency relationship between PC and MS residents or between residents and MS supervisors, when power distribution is based on inequity.
E. Fearless learning	A pattern of fearless learning is found to emerge in a safe workclimate, with collaboration being based on equity, proactively inviting each other to participate in discussions and show the courage to speak up, share perspectives and take interpersonal risks.

The themes appeared to be interacting. The observations and interviews indicated that power dynamics (the way power impacts the interaction between people) occurred both within the themes and between the themes. We described the interrelation between the themes as main types of power dynamics.

**FIGURE 1:**
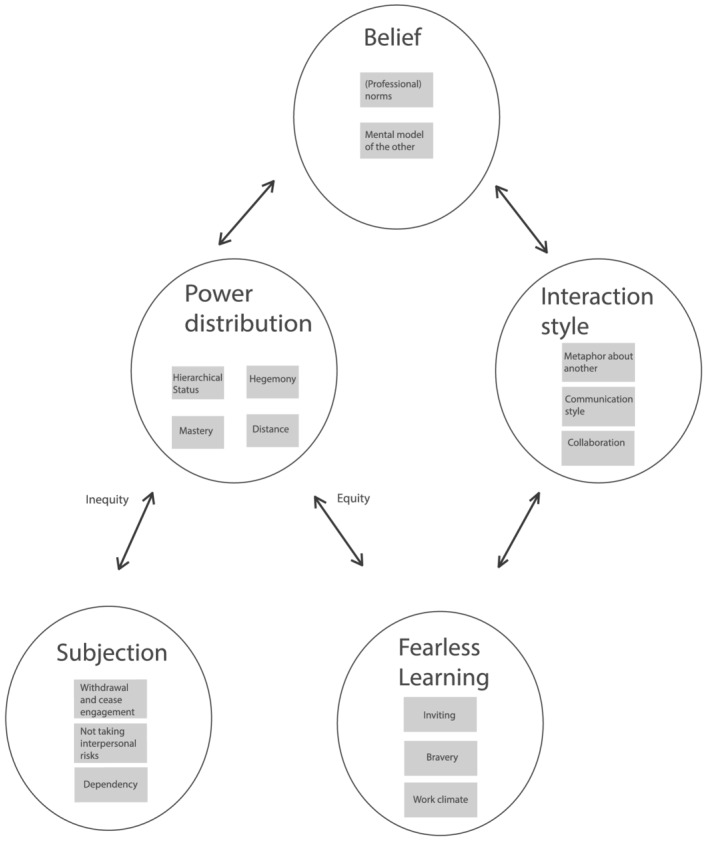
Main types of power dynamics in intraPC learning

We found five main types of power dynamics in intraPC learning between PC residents and MS residents in hospitals (see Figure 1): (i) beliefs impact power distribution; (ii) beliefs impact interaction style; (iii) power distribution based on inequity impacts subjection; (iv) power distribution based on equity impacts fearless learning; (v) interaction style impact fearless learning. We will elaborate on these themes and on power dynamics in this section.

### 4.1 Beliefs impact power distribution

Our interviews revealed that power distribution is influenced by underlying beliefs and vice versa. Supervisors mentioned that professional norms, such as mastery of knowledge, determine the level of hierarchical status assigned to PC residents.

In that case [if the PC resident has little input], he descends in hierarchy. I think that they measure this [hierarchical status] in discussions, who is saying and doing what, when and where… PC residents who dare to speak up are rewarded for that; they are heard more. MS_supervisor_D1

Supervisors and residents indicated that the beliefs they hold about each other (mental model) fuel power dynamics between PC and MS residents. PC physicians and PC residents are expected to share information for intraPC, but this is not expected of MSs and MS residents (professional norms). In order to learn intraPC, some MS residents would like to balance this inequality, but they doubt whether they have support for doing so. Several MS residents doubted whether they could learn from PC residents. These beliefs hamper the ability to learn intraPC.

There is an exchange on their side [PC physicians/residents], but conversely there is no exchange from our [MSs/MS residents] side… I do not know if people [MS (residents)] would be interested in that [exchange by MS residents], but I do think it would be important in an effort to establish proper intraprofessional care. MS_resident_D20

I'm not sure what we may learn from a PC resident… Do you have a suggestion?… I do get that PC physicians have limited diagnostics. I cannot quite imagine what we can learn directly from PC residents. MS_resident_D26

### 4.2 Beliefs impact interaction style

Our interviews demonstrated that beliefs impact interaction style, and, similarly, that the way PC residents, MS residents and supervisors talk about and with each other (often in metaphors) can create/maintain beliefs. Participants mentioned that interaction styles have a major effect on generating a constructive or unconstructive manifestation of power dynamics, which subsequently have a conducive or corrosive effect on intraPC learning (see Table 2).

**TABLE 2:** Interaction style: the way primary care (PC) residents, medical specialty (MS) residents and MS supervisors talk about and with each other, often in metaphors

Corrosive effect on intraPC learning	Conducive effect on intraPC learning
‘Handovers are a very good way to exchange experiences, to exchange learning points. […] I do miss that with surgery, but it fits with the attitude of surgeons and the attitude of internal medicine. At the internal medicine department, you are part of the team, but with the surgeons you are an accessory/a sidekick/that works along […] There is some alpha male behavior in there. Surgeons react differently if there's another specialism around. It is the kind of hierarchy I expect from a surgeon. That just belongs there. Actually, I enjoy the spectatorship, you know, I like it. I find myself gawking at their behavior.’ PC_resident_ D3	‘We assume a lot about what PC physicians can or cannot do. We have all kinds of beliefs and we naturally consider ourselves [MSs] better than PC physicians… Of course, when there is a PC resident in the group, you have to watch what you say about why you might think PCs should have done things differently… I think it's quite intimidating [for PC residents] sometimes… What I do when I notice this, is to expressly invite the PC resident to say something about it. Like, “this is happening right now, but let us ask the PC resident in our midst what he thinks about it.”’ MS_supervisor_D25
‘That I do not trust colleagues [PC residents] unless I know they are trustworthy or I witnessed it with my own eyes. You just need to have a healthy kind of suspicion, whilst having to supervise them (PC residents), to check up on them.’ MS_resident_D38	‘PC residents may think that they are a bit inferior to the work here. But really, their expertise could be of use to us as well. Since this is their hospital placement, they want to learn more about clinical geriatrics I think… Whilst it would also be great if it [discussion/exchange] could also focus on geriatrics in general practice or geriatrics in the nursing home.’ MS_resident_D20
‘Cardiology can be condescending. That really seems to be part and parcel of that specialty. … I do not think it really matters that I'm a PC resident. It's just that they are used to saying “here comes primary [emergency] care again with a stupid question”… that could affect me in terms of learning from each other, because you are less inclined to ask each other questions.’ PC_resident2_D6	‘We [MSs] often have an opinion about PC physicians. When a patient is referred too late we think: “they cannot do anything correctly, they are often incorrect, other times they missed it [a diagnosis], or acted too late. See, here we go again …” But we do not get to see everything that goes well. So we have a distorted image of their reality. We do not know the limitations they have. But by having PC residents over, you notice that we start labelling such things differently. We ask more openly, verify things with them. And so we engage with them [PC (residents)] respectfully and more constructively.’ MS_supervisor_D19

Table 2 shows that PC residents, MS residents and MS supervisors have biases and judgmental beliefs about each other, which could lead to tense interactions that impede intraPC learning. As supervisors D19 and D25 noted, awareness and recognition of beliefs could be a first step in balancing power dynamics, followed by a respectful interaction with careful language and actively inviting each other to participate in discussions.

### 4.3 Power distribution based on inequity impacts subjection

Our data indicated that power distribution between PC residents, MS residents and MS supervisors is an attribution, for example, hierarchical status due to mastery of knowledge, and can be based on systems in the medical context or organisation, for example, existing power distance between MSs and PCs or between supervisors and residents. Power distribution seemed to be an intertwining of attribution and system factors. We observed that power distribution based on inequity (hegemony) between PC and MS residents or between MS supervisors and residents shapes unconstructive power dynamics. Residents sometimes feel that the PC residents' voice does not count or is overridden. This can lead to less interpersonal risk‐taking or ceasing engagement or subjection of the PC resident, which could have a destructive effect on intraPC learning.

I would be less likely to initiate a discussion about it… I can share my PC guidelines, but they just get swept off the table. At that point I just think… fine… I'll just act submissively here and we can do this the way you want to do it. PC_resident_D14

They allowed me to tag along, so I was there to watch and to listen what this one physician was saying. And then I had to decide whether I would start a discussion to share my [PC]point of view whilst I could see that this person was not really open to it… I did not believe he was inclined to change his mind. Well, perhaps this was a bit lazy of me, but let us just leave it at that. PC_resident2_D27

Our interviews revealed that supervisors may experience the power dynamics quite differently than PC/MS residents. Our observations showed that even with a small power distance between residents, the MS resident can easily overrule the PC resident, for example, by mastery of knowledge. MS residents do not always seem to be aware of the power dynamics at play, while PC residents may be inhibited or silenced by these dynamics. This could be a barrier to intraPC learning, see Box 1.

I'm obviously at the top of the hierarchical ladder, so to what extent can someone at the top judge whether hierarchy is a factor. I do not see it as a limiting factor. MS_supervisor_D23

For [PC and MS] residents to go to their MS supervisor: that's a barrier… that certainly has to do with hierarchy. MS_resident_D33

**Box 1: Two examples of education at the workplace (hospital departments)**

*Shockroom training (simulation) with MS residents, PC residents, nurses and undergraduate students; teaching was prepared by a couple of a MS resident and a PC resident*:
*It seems that the MS residents mainly educate the others. The atmosphere is relaxed and based on equity. After the simulation, a student asks the PC resident what he would do if this patient showed up in general practice. PC resident does not seem to get a chance to answer this question and is overruled by an MS resident who immediately gives an answer, complemented by another MS resident. A moment later, another intern asks the PC resident why the patient was so agitated in this case. Two MS residents answer this question directly. Again, the PC resident does not seem to get an opportunity to answer for himself, although the question comes directly to him. This hampers the chance of intraPE*.
**Fieldnote_R1_R2_H1**
*Joint teaching session (12.30): at the start, 1 PC resident, 6 MS residents and 2 undergraduate students attend. They are discussing a patient case. The atmosphere is relaxed and the hierarchy feels rather flat. After 20 min, a supervisor joins the session. Almost immediately after sitting down, the supervisor comments on the case study about symptoms displayed on the screen. This is followed by a discussion between 3 MS residents. At 12.55 two more supervisors join the session. They recognise the patient on the screen and immediately get involved in the discussion. The atmosphere is still relaxed but the hierarchy feels less flat than before the three supervisors joined the group. The supervisors intervene quickly and often in the discussion and take over the lead and the residents become more and more silent, sharing their perspectives less and less*.
**FieldnoteR1_H3**

### 4.4 Power distribution based on equity impacts fearless learning

A prevailing view among participants is that a certain degree of hierarchical power distribution in the medical workplace can contribute to a constructive manifestation of power dynamics. As long as collaboration is based on equity, hierarchical power distribution could foster a work climate that contributes to fearless intraPC learning during hospital placements. As the following residents said:

There is a hierarchy, but everyone can quite easily contact each other. It's clear who's ultimately responsible. They're not vague about it because that would actually hinder a good working atmosphere. That [collaboration] just occurs in a very relaxed way. PC_resident_D40

We stand above PC residents, but not in rank or anything. It's more that you are really above them in terms of knowledge, but not in how you treat each other or whatever… Look, a PC resident may not treat a neurotrauma, that's a difference of course. It does not make me feel better or higher. MS_resident_D7

Our observations and interviews suggest that equity can be promoted by sharing a physical space in which everybody literally stands or sits at the same level during patient discussions.

Previously, we were hierarchically separated in the handover room, but we made a conscious decision to have everyone on the same level during the handover, just to be able to discuss everything face‐to‐face with each other. MS_supervisor1_D5

I think that's also one of the reasons that the day‐start is always done standing up, so that everyone is equal. PC_resident2_D35

### 4.5 Interaction style impact fearless learning

Participants indicated that open interactions enable fearless intraPC learning because residents and supervisors feel the bravery to speak up in open interactions. Some supervisors noted, therefore, that they are attentive to asking open questions (collaboration, inviting):

Then [asking open‐ended questions] you get much more discussion, much more. It's also much safer… That's why we pay so much attention to it. And when the department head is a bit adamant, that's annoying. Then it's done, and everyone keeps quiet. Yes, that kills the discussion and decreases the [intraPC] learning effect… We know by now how big the consequences are, so we are very careful about that. MS_supervisor_D1

**Box 2: Handover based on open interaction at the geriatrics department**

*The handover room is an uncluttered area with three posters on the wall. One poster lists conversation rules*:
*Handover discussion rules*:
*‐Let each other talk and listen to each other's arguments*
*‐Be open to each other's opinions*
*‐Remain rational and fight arguments based on content*
*‐Discuss on the basis of equality*
*The posters are there as a reminder, and it is noticeable that people comply with these rules, as can be seen in the interaction below*:
*Three supervisors discuss the admission of a patient to Medium Care (MC), and this patient is bedridden and may need to be admitted to a nursing home with more care. A PC resident joins the discussion non‐verbally (nodding, shaking, frowning* etc.*) before saying: ‘This is a fragile patient who cannot make decisions for herself; she has no overview and was already bedridden before admission. Maybe it's my PC perspective, but I'd say: where's the gain in this [admission to MC]? You're not going to do all that, are you?’ Supervisor 3 says ‘This is indeed a cascade, and I recommend consulting the general practitioner first. MC is not a meaningful option: it has no medical benefits, and so we should indeed not suggest that.’ With input from the PC resident, the plan was adjusted from MC to consultation (intraPC) with general practitioner*.
**Fieldnote_R1_H2**

Participants mentioned that MS supervisors can play an important role in managing power dynamics and creating a safe work‐climate for intraPC learning. To promote fearless learning, some supervisors indicated that they have made policy changes to create a speak‐up culture. One supervisor gave an example of an active policy against unconstructive impact of power dynamics at their department:

We have a very clear speak up‐culture in our department. That has grown over the last years. Everyone treats each other with respect. We find that extremely important. If you do not, you are really put back in your place here. And that goes for both residents and bosses. To cite an example, two years ago, a colleague [MS] was barking at a resident in the hallway. And the emergency room doctor here told him, ‘You'll never do that again, or I'll have you fired on the spot.’… There should be no threshold for consultation. MS_supervisor1_D5

Another way to promote fearless intraPC learning in the hospital ward is to start the workday or team meeting with a personal briefing or by registering a smiley face that reflects the person's mood. Participants indicated that sharing thoughts, feelings and learning goals could support the connection between team members and balance power dynamics.

Yes, we consciously chose this [as a start to team‐meetings] because studies have shown that employees feel more valued and you also get better team bonding when you first pay attention to whether everyone is fit and if there's anything we need to take into account. MS_supervisor1_D42

## 5 DISCUSSION

Many calls have been made in previous studies to examine and address the influence of power on intraprofessional learning.^30^, ^31^, ^45^ To our knowledge, this is the first study specifically investigating power dynamics and their impact on intraPC learning between PC and MS residents during hospital placements. Our data showed five themes that describe characteristics of power dynamics: (i) beliefs; (ii) power distribution; (iii) interaction style; (iv) subjection; (v) fearless learning. These themes were found to be interrelated, and power dynamics among residents and/or supervisors occur both within and between the themes. We report five main types of power dynamics in intraPC learning between PC and MS residents in hospitals: (i) beliefs impact power distribution; (ii) beliefs impact interaction style; (iii) power distribution based on inequity impact subjection; (iv) power distribution based on equity impact fearless learning; (v) interaction style impact fearless learning.

### 5.1 Beliefs and interaction

Our data suggest that beliefs feed into power and into the way professionals talk about and with each other, and that the nature of the interaction, conversely, create/sustain beliefs, both at the individual and the group levels. Our findings are in line with previous studies in other fields, such as organisational psychology and neuroscience, showing that all types of interactions have emotional subtexts^50^ and are contagious,^50^, ^51^, ^52^, ^53^, ^54^, ^55^ a form of social influence in which individuals directly alter each other's brain activity,^50^, ^51^ attitudes, cognitions, emotions and behaviours.^50^, ^52^, ^53^, ^56^

Such contagion has a profound effect on power dynamics, collaboration quality^53^ and team outcomes.^51^, ^52^ We found that expressing negative beliefs and attitudes about another profession could lead to an unconstructive manifestation of power dynamics that negatively impact intraPC learning. At the same time, our data indicate that changing the form of interactions by consistently applying conversation rules or other regulations could already have a transformative effect on intraPC (learning) in hospitals as it opens the door to candid discussions. Prior studies demonstrate that the contagiousness of positive interactions, based on curiosity, trust, dignity and confidence,^50^, ^51^, ^57^ can lead to better collaboration,^51^, ^53^ better learning^51^ and fewer conflicts.^54^ A powerful first step in changing the impact of power dynamics is to change how we talk. This stresses the importance of residents and supervisors being aware of their attitudes and beliefs and the way they express themselves, and recognising which type of interaction encourages intraPC (learning), making the implicit explicit.

### 5.2 Interaction style and fearless learning

This study indicates that a constructive manifestation of power dynamics can occur when hierarchical power distribution is combined with open interactions and collaboration based on equity. This is consistent with prior research revealing an inextricable link between open interactions and psychological safety.^42^ In contrast, we found that a lack of equity and open interactions, for example, when PC residents feel that their voices do not count or are overruled, can lead to their ceasing engagement or subjection, which is detrimental as sharing perspectives and speaking up are essential for intraPC learning. If open interactions were to be applied as merely a technical skill without really being prepared for discussion, the underlying biases and attitudes will still create power dynamics.

Although PC residents may be obstructed by power dynamics, our study shows that supervisors and MS residents are not always aware of the impact of these dynamics being at play. Even with the power distance between residents being small, MS residents could easily and unintentionally overpower PC residents. One possible explanation for this is the interrelation between hierarchical status and perceived psychological safety^7^: higher‐status MS residents appear to feel safer and hence more comfortable speaking up^41^ than lower‐status PC residents.

A powerful way to foster psychological safety and fearless learning is by acknowledging each other's opinion,^56^, ^58^ by sharing mutual attention^50^ and by actively reducing inequity.^41^, ^56^ This study yields practical suggestions on how this can be done between PC and MS residents and supervisors: purposefully inviting each other to participate in discussions, asking open‐ended questions, being open to other perspectives and criticism, having a functional distribution of power roles combined with consultation based on equity and consistently sharing thoughts and feelings in a personal briefing during team meetings.

### 5.3 Fearless learning in action

As healthcare and residency training have a strongly hierarchical nature with associated strong professional norms,^5^, ^7^, ^59^, ^60^ sustaining fearless intraPC learning on the hospital ward could be easier said than done.^60^ Previous studies suggested the need for a profound cultural change to enforce psychological safety and fearless learning,^42^, ^61^ the need for identifying specific supervisor behaviours that can minimise power dynamics, and the need for shaping interventions and organisational changes that will cultivate fearless learning among residents^7^, ^8^, ^60^ on the hospital ward. This study, however, indicated that effective change could already be achieved by smaller interventions that are quite easy to implement. Participants noted that supervisors can play an important role in managing power dynamics for the purpose of fearless intraPC learning and participants mentioned various policy changes to balance power dynamics and to support fearless intraPC learning (see Section 5.4).

### 5.4 Implications for practice

To manage power dynamics and to facilitate fearless intraPC learning between residents in hospitals, the following ideas might be helpful: (i) Invite each other purposefully into discussions and be attentive to listening and asking open‐ended questions as a team. Put a poster on the wall with clear conversation rules and (if necessary) consistently remind each other of these agreements during team meetings; (ii) implement an active policy of treating everyone with respect and counteracting unequal power dynamics. Talk to each other about disruptive behaviour; (iii) share physical spaces in which people literally stand or sit at the same level during team meetings; (iv) start workdays or meetings with a personal briefing or have staff register emotions by selecting a smiley face that reflects someone's mood; (v) be aware of the beliefs and the way residents and supervisors talk with and about each other and recognise which type of interaction encourages intraPC (learning), making the implicit explicit; (vi) distribute power roles and responsibilities functionally and collaborate on the basis of equity.

Representing the residents' and supervisors' perspective is important for understanding the influence of power dynamics on intraPC learning between residents in hospitals, and it becomes crucial when the goal is to balance these power dynamics in order to foster fearless intraPC learning. This study describes a phenomenon that is often more implicit than explicit; however, this study also demonstrates that not all beliefs, biases and practices are ‘hidden’; some are perceptible, taken for granted and part of the traditional culture passed down to the next generation. Collaboration during postgraduate training sets the tone for quality of future intraPC. IntraPC learning goes beyond learning new skills and empowering residents, it is a matter of creating a culture of sincere equal collaboration. A deeper understanding of power dynamics and their impact could be useful to open the door to culture change and to further improve intraprofessional collaboration.

### 5.5 Limitations

We recognise that there may be more types of interactions between the themes, for example between beliefs and fearless learning or between interaction style plus power distribution that may promote subjection, but these did not emerge from our study. Some interviewees were very open about power struggles, while others were holding back. As this research was part of a larger project which had a broader scope than power dynamics alone, we may have missed depth or an opportunity to break through interviewees' hesitations. As the analysis shows data saturation, however, we feel confident about our results.

Triangulation with observations, moreover, helped to gain insight into who were holding back and to get ideas about why this might be the case or what was actually happening in the workplace. Still, it is important to remember that power is a taboo subject, and it may have been difficult for interviewees to really speak up.

### 5.6 Future research

Further research is needed to determine whether and how the listed implications for practice will help to improve fearless intraPC learning. Future studies could focus on using a phenomenological approach in the interviews to really understand the interviewees' perspective. As the topic of power dynamics remains a taboo subject, we recommend focusing on trust before the interview and including metaphors to get an idea of actual beliefs. Based on our experience, we recommend triangulation with observations, because this could be helpful in understanding whatever is not mentioned in interviews.

## 6 CONCLUSION

Power dynamics have an impact on intraPC learning between residents in hospitals. Power distribution between PC residents, MS residents and MS supervisors seems to be an attribution affected by underlying beliefs about professional norms or about other professions. Beliefs influence the way PC residents, MS residents and supervisors interact. Power distribution based on inequity could cause PC residents to be subjected, and power distribution based on equity could lead to fearless learning. Open interactions enable interconnection and fearless intraPC learning. We conclude that the manifestation of power dynamics could be constructive for intraPC learning during hospital placements if power distribution is based on equity, combined with sincere open interactions, actively inviting each other into discussions and enlisting the support of MS supervisors to foster fearless intraPC learning. This can be achieved by creating awareness of implicit beliefs and by making them explicit, recognising interaction that encourages intraPC learning and creating policies that support fearless intraPC learning.

## CONFLICTS OF INTEREST

No competing interests.

## ETHICAL APPROVAL

We asked the NVMO (Netherlands Association for medical Education) Ethical Review Board for ethical review of our research proposal at 15 December 2017. The NVMO Ethical Board approved the study on 15 February 2018, dossier number 983.

## AUTHOR CONTRIBUTIONS

Natasja Looman met all of the following conditions: substantial contributions to the conception and design of the work; the acquisition, analysis, and interpretation of data for the work; AND drafting the work and revising it critically for important intellectual content; AND final approval of the version to be published; AND agreement to be accountable for all aspects of the work in ensuring that questions related to the accuracy or integrity of any part of the work are appropriately investigated and resolved.

Tamara van Woezik met all of the following conditions: substantial contributions to the conception and design of the work; the analysis, and interpretation of data for the work; AND drafting the work and revising it critically for important intellectual content; AND final approval of the version to be published; AND agreement to be accountable for all aspects of the work in ensuring that questions related to the accuracy or integrity of any part of the work are appropriately investigated and resolved.

Dienek van Asselt met all of the following conditions: substantial contributions to the conception of the work; the acquisition and interpretation of data for the work; AND drafting the work and revising it critically for important intellectual content; AND final approval of the version to be published; AND agreement to be accountable for all aspects of the work in ensuring that questions related to the accuracy or integrity of any part of the work are appropriately investigated and resolved.

Nynke Scherpbier met all of the following conditions: substantial contributions to the conception and design of the work; the acquisition, analysis, and interpretation of data for the work; AND drafting the work and revising it critically for important intellectual content; AND final approval of the version to be published; AND agreement to be accountable for all aspects of the work in ensuring that questions related to the accuracy or integrity of any part of the work are appropriately investigated and resolved.

Cornelia Fluit met all of the following conditions: substantial contributions to the conception and design of the work; the acquisition, analysis, and interpretation of data for the work; AND drafting the work and revising it critically for important intellectual content; AND final approval of the version to be published; AND agreement to be accountable for all aspects of the work in ensuring that questions related to the accuracy or integrity of any part of the work are appropriately investigated and resolved.

Jacqueline de Graaf met all of the following conditions: substantial contributions to the conception and design of the work; the analysis, and interpretation of data for the work; AND drafting the work and revising it critically for important intellectual content; AND final approval of the version to be published; AND agreement to be accountable for all aspects of the work in ensuring that questions related to the accuracy or integrity of any part of the work are appropriately investigated and resolved.

## Data Availability

The data that support the findings of this study are available from the corresponding author upon reasonable request.
